# Formation and characterization of non-growth states in *Clostridium thermocellum*: spores and L-forms

**DOI:** 10.1186/1471-2180-12-180

**Published:** 2012-08-16

**Authors:** Elizabeth B Mearls, Javier A Izquierdo, Lee R Lynd

**Affiliations:** 1 Thayer School of Engineering, Dartmouth College, Hanover, NH, 03755, USA; 2 BioEnergy Science Center, Oak Ridge National Laboratory, Oak Ridge, TN, 37831, USA; 3 Present Address: Center for Agricultural and Environmental Biotechnology, RTI, Research Triangle Park, 27709, USA

## Abstract

**Background:**

*Clostridium thermocellum* is an anaerobic thermophilic bacterium that exhibits high levels of cellulose solublization and produces ethanol as an end product of its metabolism. Using cellulosic biomass as a feedstock for fuel production is an attractive prospect, however, growth arrest can negatively impact ethanol production by fermentative microorganisms such as *C. thermocellum*. Understanding conditions that lead to non-growth states in *C. thermocellum *can positively influence process design and culturing conditions in order to optimize ethanol production in an industrial setting.

**Results:**

We report here that *Clostridium thermocellum* ATCC 27405 enters non-growth states in response to specific growth conditions. Non-growth states include the formation of spores and a L-form-like state in which the cells cease to grow or produce the normal end products of metabolism. Unlike other sporulating organisms, we did not observe sporulation of *C. thermocellum* in low carbon or nitrogen environments. However, sporulation did occur in response to transfers between soluble and insoluble substrates, resulting in approximately 7% mature spores. Exposure to oxygen caused a similar sporulation response. Starvation conditions during continuous culture did not result in spore formation, but caused the majority of cells to transition to a L-form state. Both spores and L-forms were determined to be viable. Spores exhibited enhanced survival in response to high temperature and prolonged storage compared to L-forms and vegetative cells. However, L-forms exhibited faster recovery compared to both spores and stationary phase cells when cultured in rich media.

**Conclusions:**

Both spores and L-forms cease to produce ethanol, but provide other advantages for *C. thermocellum* including enhanced survival for spores and faster recovery for L-forms. Understanding the conditions that give rise to these two different non-growth states, and the implications that each has for enabling or enhancing *C. thermocellum* survival may promote the efficient cultivation of this organism and aid in its development as an industrial microorganism.

## Background

*Clostridium thermocellum* is an anaerobic soil bacterium that is of particular interest due to its complexed cellulase system and its ability to rapidly solubilize cellulosic material and produce ethanol [1]. Like all clostridia, this organism forms terminal endospores, which confer a high degree of resistance to heat, desiccation and other environmental challenges. Understanding sporulation and other non-growth states from a fundamental perspective is also relevant to culture management and performance in applied contexts.

In bacteria, dormant or non-growth states have been defined as “a reversible state of low metabolic activity in a unit which retains viability” [2]. Bacterial spores are produced by Gram-positive bacteria including members of the *Bacillus* and *Clostridium* genera, and are widely understood to be dormant cell forms that remain viable for long periods of time until growth conditions become favorable. In well-studied *Bacillus* species, factors inducing spore formation include the end of exponential growth, a decrease in dilution rate during continuous culture, and limitation by carbon or nitrogen [3,4]. In *Clostridium perfringens*, sporulation is triggered by low pH, inorganic phosphate, the presence of complex polysaccharides, and possibly a quorum sensing mechanism at high population densities[5,6]. However, the impact of nutrient limitation on sporulation has not been conclusively determined in *C. perfringens* or other pathogenic *Clostridia*[5]. *Clostridium acetobutylicum*, a non-pathogenic solventogenic organism, also initiates sporulation at low pH, but not in response to carbon or nitrogen limitation [7].

Spore formation is less well-studied in cellulolytic organisms. Most of the work on sporulation in cellulolytic clostridia has been done with *Clostridium cellulolyticum* in which increased spore formation resulted from carbon starvation during exponential growth [8], growth at low dilution rates [9,10], ammonium limitation [9], low pH, and the presence of insoluble substrate [10]. Spore formation has previously been reported in *C. thermocellum* strain JW20 [11,12], for which spore formation occurred once the pH had dropped below 6.4. Freier et al. also noted spore formation after the temperature dropped below 48 °C and that growth on cellulose seemed to enhance the sporulation response to a greater extent than growth on other substrates. Spore formation has not been evaluated for strains of *C. thermocellum* other than strain JW20, which was determined to be a co-culture of *C. thermocellum* and the non-spore forming* Thermoanerobacter ethanolicus*[13]. In particular, spore formation has not to our knowledge been evaluated in strain ATCC 27405, which has been widely studied with respect to both physiology [1,14-16] and properties of its cellulosome enzyme system [15-19].

L-forms have been observed in a variety of bacterial species, including *Clostridium* species other than *C. thermocellum,* after exposure to different stressors. The term L-form is often used interchangeably with other terms such as CWD-forms (cell-wall-deficient), L-phase, L-variants, autoplasts, cysts, round bodies, spheroplasts, and protoplasts [20-22]. The metabolic activity of L-form bacteria has not been widely studied, but previous work has shown that metabolic activity for the L-form is often much lower than vegetative cells [23,24]. Generally L-forms can be recognized by a spherical or pleomorphic morphology which differs significantly from the morphology of the parent cells [25], but as the shape of L-forms can vary considerably, this definition is not universal. They are most frequently defined as cell forms that have a deficient or absent cell wall and retain the ability to divide [26]. The ability of L-forms to form colonies on nutrient rich plates [26] helps to differentiate them from viable but non-culturable cells (VBNCs), another non-growth state which is often induced by starvation or unpermissive growth temperatures and in some cases shares many similar features with L-forms [27]. L-forms are often classified in two categories, stable and unstable, which respectively refer to whether the L-form can revert back to the parent morphology or not [21]. Stressors that have been found to induce or promote the L-form morphology include treatment with β-lactam antibiotics with or without lysozyme[28,29], cultivation in minimal media or exposure to nutrient limitation [30-32], exposure to extreme heat [30] and exposure to high salt concentrations [33].

Following the observation that *C. thermocellum* strain ATCC 27405 develops L-forms in addition to spores, we examine here the properties of these two non-growth cell states and the factors that trigger their formation in this organism.

## Results

### Evaluation of conditions under which spores were observed

Several growth medium modifications were tested to evaluate impacts on sporulation of *C. thermocellum* strain ATCC 27405 as shown in Table 1. Only the absence of vitamins appeared to have any sporulation effect, with an average of 4% of the cells forming spores. Elevated amounts of acetate (3 g/L) and ethanol (0.2-10% v/v), the two primary fermentation products formed by this organism, were also tested but a sporulation response was not observed. The effect of low pH was tested in *C. thermocellum* cultures allowed to drop below pH 6.0 during the course of normal fermentation, but sporulation was not observed. Likewise, a decrease in temperature below 48°C did not result in spore formation for exponential or stationary phase cells.

**Table 1:** Percentage of resting cells formed after stress exposure

**Stress type**	**Specific modification**	**Percent spores**	**Percent L-forms**
MTC media (control)	No modifications	0	0
Nutrient limitation	Reduced cellulose (1g L^-1^)	0	0
Nutrient limitation	Low phosphorous	0	0
Nutrient limitation	Low nitrogen	0	0
Nutrient limitation	No vitamins	4.2 ± 2.8	0
Inhibitor	Added ethanol	0	0
Inhibitor	Added acetate	0	0
Oxidative stress	Added oxygen	6.6 ± 4.0	0
Substrate change	Transfer from cellulose to cellobiose	6.9 ± 3.7	0
Substrate changes	Transfer from cellobiose to cellulose	6.2 ± 3.7	0
Starvation	Depletion of substrate during steady state growth	0	98.0 ± 0.017

Conditions predicted to be unfavorable for growth were tested to determine which stressors cause *C. thermocellum* to form spores or L-forms. The percentage of resting cells to total cells is shown. Error represents one standard deviation, n = 3.

**Figure 1:**
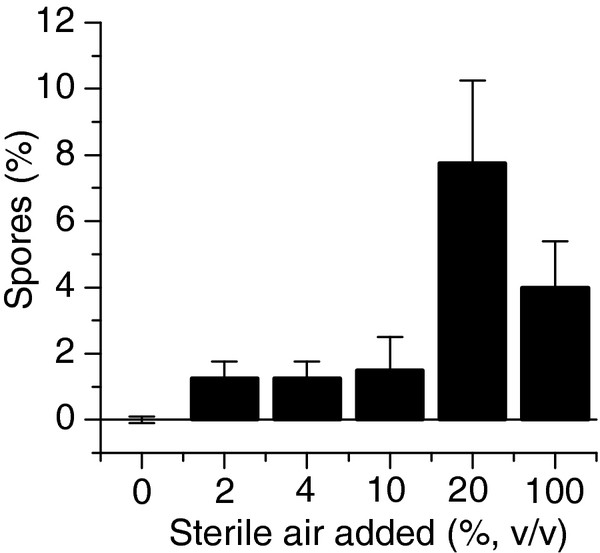
**Sporulation induced by aerobic cultivation.** The effects of oxygen on spore formation were determined by exposing *C. thermocellum* cultures to increasing volumes of sterile air. Error bars represent one standard deviation, n = 3.

Conditions that resulted in sporulation included oxygen exposure and changes between growth on soluble and insoluble substrates. As *C. thermocellum* is an obligate anaerobe, oxygen was chosen as a stressor. Varying amounts of oxygen were tested and as is shown in Figure 1, the addition of 20% v/v sterile air to the headspace of a sealed serum vial grown culture was optimal for inducing spore formation. Oxygen induced spore formation in approximately 7% of the cells. Additionally, approximately 7% of the cells sporulated when transferred from cellobiose to Avicel or from Avicel to cellobiose (Table 1). *C. thermocellum* can grow equally well on both substrates, and when cultures are transferred or subcultured in media with the same substrate, sporulation was not observed. L-forms were not observed in any of the conditions mentioned above.

### Evaluation of conditions under which L-forms were observed

**Figure 2:**
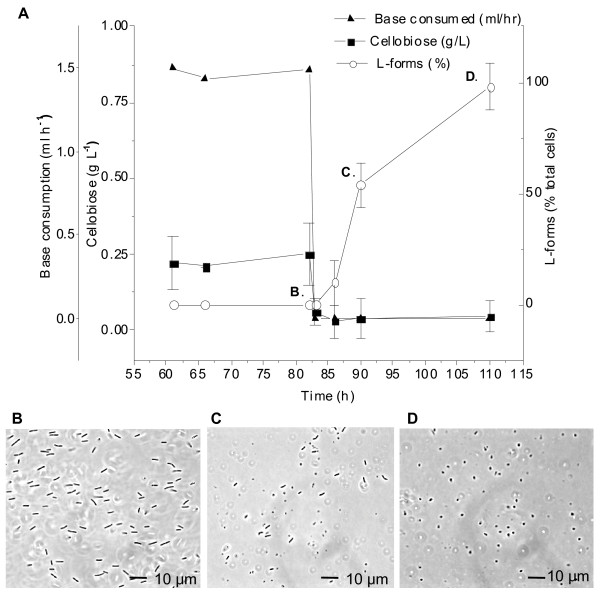
**L-form induction occurs after cellobiose depletion.A**) After steady state growth is established (T = 60-82 h), the growth rate remains constant, as is indicated by the concentration of cellobiose (■) and the rate of base addition (▴). At 82 h, continuous feed is stopped and the rate of base addition decreases to 0 ml/h while the remaining cellobiose is entirely consumed. The percentage of L-forms (○) present in the culture increases steadily after the feed is stopped until nearly all cells have transitioned. **B**) Cells at 82 hr, just before the feed is stopped. **C**) Cells at 90 hr (8 hours after the feed is stopped), L-forms begin to form.** D**) Cells at 110 hr (28 hours after the feed is stopped), only L-forms are observed in the culture. Error bars represent one standard deviation, n = 3.

**Figure 3:**
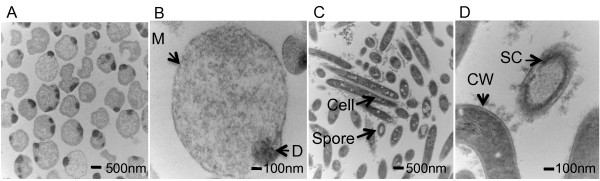
**TEM images of L-forms, spores and cells.** TEM was used to obtain images of L-forms, spores and cells to compare their morphology and structure. The L-form population lacks a cell wall resulting in spherical or pleomorphic cell morphology (Figure 3**A** and 3**B**). The cell membrane (M) is visible, and in many cases, a dark protrusion (D) of unknown function is observed (3**B**). Images of cells clearly show the cell wall (CW), and *C. thermocellum’s* normal rod morphology (Figure 3**C** and 3**D**). Coccoid-looking cells in Figure 3**C** are indicative of cells that were cross-sectioned across their diameter, but the cell wall structure is still easily recognized. The spore coat (SC) is also easily recognized as a several dense layers (Figure 3**D**).

Abrupt termination of the feed to a steady-state continuous culture at several dilution rates (0.03 h^-1^, 0.1 h^-1^, and 0.15 h^-1^) and with several cellobiose concentrations (2.5, 3.0 and 5.0 g/L) was used to evaluate the impact of sudden substrate exhaustion in *C. thermocellum*. This treatment, independent of dilution rate or cellobiose concentration, was found to cause nearly all of the cells to shift to the L-form morphology (Table 1, Figure 2) with no spores observed. L-forms were readily distinguished from spores by light microscopy, appearing phase dark and nearly translucent whereas spores are phase bright and opaque. Further analysis by TEM clearly showed structural differences between L-forms and spores (Figure 3). We, as well as others [11], have observed *C. thermocellum* spores to exhibit a thick spore coat (Figure 3C and 3D), whereas the L-form cells appeared to lack a cell wall (Figure 3B) and often exhibited dark protrusions (Figure 3A and 3B). Essentially all cells following substrate exhaustion in continuous culture exhibited transition to the L-form cell type. This is in contrast to the sporulation responses observed, in which complete spore formation was never above 10% of the total cells under any of the conditions tested.

During normal cultivation of *C. thermocellum*, L-forms are occasionally observed, but the clear transition rapidly following termination of feeding in continuous culture seemed to indicate a well-defined physiological response. Arrest of growth and metabolism following feeding termination was confirmed by HPLC analysis, showing that cellobiose was exhausted within 60 minutes and by the simultaneous cessation of base addition used for pH control (Figure 2, Panel A). No additional acetic acid, lactic acid, or ethanol was produced during this transition or after L-form formation (data not shown). The complete transition into the L-form morphology occurred approximately 24 h after the feed was stopped (Figure 2, Panel D). Once the transition from rods to L-forms was complete, viability was determined by plating. Viable counts indicated that 10^8^ CFU/ml cells remained viable in the culture at this initial time point, but that viability decreased with age (data not shown). The resulting colonies exhibited normal morphology, and all cells within the colonies were rod shaped when examined microscopically. This suggests that these L-forms were unstable, and able to revert back to the normal morphology once sufficient nutrients were supplied. To be certain the culture was free of contaminants, 16S rRNA gene sequencing was performed on several single colonies obtained, and no such contaminants were found.

### Determination of heat tolerance

**Figure 4:**
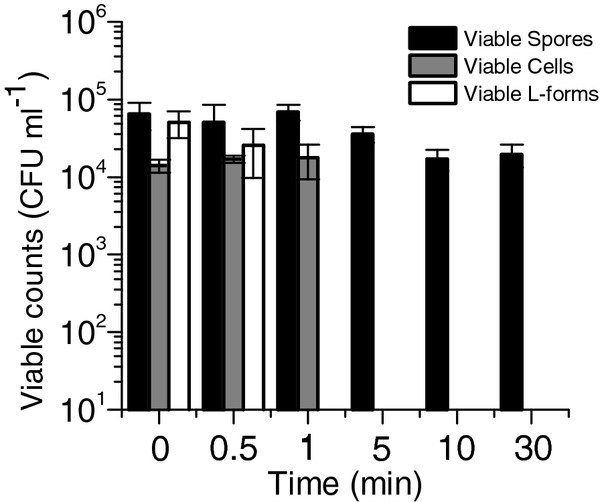
**Heat Stress Tolerance.** The ability of each cell type to tolerate heat stress was tested by exposing all cell types to100°C for 0–30 minutes. Results reported are a measure of viable counts after heat treatment. The lower limit of detection was 10 CFU ml^-1^. Error bars represent one standard deviation, n = 3.

Tolerance to 100°C was evaluated for preparations of spores, rod-shaped vegetative cells, and L-forms. As shown in Figure 4, spores tolerated boiling for 30 minutes (the maximum time tested) without a significant decrease in viability, whereas L-forms and vegetative cells could only tolerate 30-60s of treatment before losing viability.

### Dynamics of growth recovery

**Figure 5:**
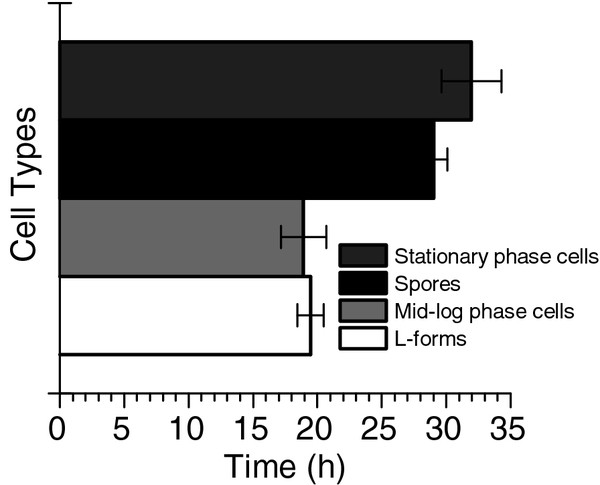
**Lag time for different cell types.** The growth recovery of spores and L-forms was compared to normal cells by observing the time required for each cell type to reach OD 0.1, and thus end lag phase. Three biological replicates are represented showing the respective lag time for each cell type. Error bars represent one standard deviation, n = 3.

In order to compare the dynamics of growth recovery, preparations of spores, rod-shaped cells, and L-forms initially at 10^3^ CFU/ml were grown in a spectrometer with OD_600nm_ readings collected every three minutes. Three separately generated populations of L-forms, three separate stocks of spores, and three independently grown cultures of cells in exponential or stationary growth phase were used for comparison. To determine the time required for each cell type to recover and resume growth, we measured the time it took for each culture to reach an O.D. of 0.1, which we take to be representative of the end of lag phase and the beginning of exponential growth. Populations of L-forms resumed growth between 18.5 and 20.5 h, exponentially grown cells between 18 and 21 h, spores between 28 and 30 h, and stationary phase cells between 30 and 34 h (Figure 5).

## Discussion

In this study, we characterized the effect of several stressors on *C. thermocellum.* Our results show that *C. thermocellum* is generally tolerant of many of the stressors that it was exposed to, such as low phosphorous, low nitrogen, and added inhibitory substances such as acetate and ethanol. *C. thermocellum* was less tolerant of vitamin deficiency, exposure to oxygen and changes in the types of available carbon source, each of which triggered spore formation. The sporulation response observed as a result of alternating carbon source between cellobiose and Avicel was surprising, as *C. thermocellum* can grow equally well on each. One possible explanation for this effect may be that *C. thermocellum* produces a large protein complex, known as the cellulosome, which acts to break down insoluble substrates [17]. The cellulosome is important for growth on cellulose, and its constituent parts are expressed at lower levels when *C. thermocellum* is grown on soluble substrates such as cellobiose [17,19,34]. The change in enzyme requirements and production after a change in substrate may induce enough stress to cause a sporulation response, as was observed in this study.

The response leading to the L-form resting state was more dramatic than the response that lead to spores, with approximately 98% of the cells in the culture transitioning to the L-form state when starved of cellobiose. It is interesting to note that L-forms were not observed at the end of a continuous cellulose fermentation (data not shown), indicating the dramatic exhaustion of available substrate may be an important trigger for L-forms. Once in the L-form state, no growth was detected by base addition, optical density, or viable counts, and end-product analysis via HPLC indicated no further production of ethanol, acetic acid, or lactic acid, the normal endproducts of *C. thermocellum* metabolism. However, L-forms did remain viable at 10^8^ CFU/ml at the time of formation. Additionally, once L-forms were inoculated into new media with adequate carbon source, they resumed growth as normal rod-shaped cells. The most cited definition of L-forms defines them as an alternative growth state [26]. This is because in some cases L-forms are able to divide by a process similar to budding [25,35], or via reproduction within the L-form and subsequent release after the lysis of the mother cell [36]. Reproduction of L-forms was not observed in *C. thermocellum* cultures, though many of the L-forms did have small dark protrusions, previously observed and hypothesized to be budding cells in *Haemophilus influenzae* L-forms [37], but never conclusively determined to be such [21]. Quantification of *C. thermocellum* L-forms over time to determine how many persisted in culture indicated only decreases in cell population over time (data not shown), indicating cell death, not proliferation. However, because *C. thermocellum* L-forms are induced by severe nutrient limitation, it is difficult to assess their ability to grow and divide as the necessary nutrients needed to promote normal growth are absent during *C. thermocellum* L-form formation and cultivation.

Traditionally, most lab-cultured L-forms are induced by treatment with antibiotics that target the cell wall. In this case, cells may escape the deleterious effects of this treatment by transitioning to the L-form state. This has been proposed as a method for pathogenic organisms to survive in a host in spite of antibiotic treatment [38,39]. However,the development of L-forms is not limited to antibiotic treatments. Other authors have postulated that the L-form state constitutes a means for cells to escape an unfavorable growth environment [30,32] or as a biologically relevant state in non-lab environments [33]. In Markova et al., *E. coli* L-forms were seen to form after exposure to extreme heat stress, and after prolonged cultivation in minimal media. Several accounts of *Borrelia burgdorferi* L-forms (also referred to as cysts or round-bodies) were observed after starvation conditions [31,32], in which serum-minus media and water were each used for induction. In one such study, Alban et al. determined that once sufficient nutrients were supplied, up to 52% of the L-forms were able to revert back to normal cells, though this number decreased with increasing age of the culture. These instances of L-form induction and recovery closely mirror what we observe in *Clostridium thermocellum*. The destruction of the cell wall, or the failure to maintain it, may be representative of a cell struggling to keep or obtain the energy needed for survival.

Once we determined that *C. thermocellum* L-forms were viable, we questioned why the cells would form an L-form rather than remain rod-shaped or form a spore. It seemed unlikely that L-forms were deformed or unformed spores, as defects in spore formation manifest in identifiable stages, none of which resemble the L-form. We therefore hypothesized that L-form formation provided some advantage for *C. thermocellum.* One potential explanation is that transitioning to an L-form requires less energy than sporulation or conserves energy overall for the cell. It is also possible that L-forms provide some advantage over spores or rod-shaped cells in terms of survival or recovery. Testing the first scenario effectively would have been technically difficult, so we went about testing the second hypothesis.

To compare spores, rod-shaped cells, and L-forms in terms of survivability and recovery, we tested how well each cell type tolerated heat and how quickly each could resume growth. *C. thermocellum* spores proved to be much better at tolerating heat stress than L-forms or rod-shaped cells suggesting advantages for *C. thermocellum* spores in prolonged survival under other stressful conditions. L-forms did not survive heat stress as well as spores, but did exhibit a shorter lag-phase upon recovery when compared with both spores and stationary phase cells, each of which took over 9 hours longer to begin exponential growth. While L-forms demonstrated faster recovery, L-form viability over time was consistent with that of stationary phase cells when subjected to prolonged starvation. This suggests that the primary advantage for *C. thermocellum* in forming an L-form does not lie in enhanced viability over time, but rather in the ability to recover rapidly when conditions become favorable for growth. This feature may allow for L-from cells to out-compete other non-growing cells in natural environments.What molecular or physiological triggers come into play to determine whether a cell becomes spore, an L-form or remain rod shaped remain to be explored.

## Conclusions

In this work we were able to define conditions that gave rise to either spores or L-forms in *C. thermocellum* ATCC 27405. Of particular interest is the formation of spores in response to changes in substrate. This result suggests that *C. thermocellum* has a preference for continued cultivation on one substrate and variations in substrate supplied during cultivation may need to be minimized in order to optimize growth. To our knowledge this is the first documentation of the L-form state in *C. thermocellum*, and the first comparison between spores and L-forms in one organism. Under no conditions were both resting cell types observed simultaneously, suggesting that these two states are unlikely to be linked at the molecular level, though further study is warranted. While the formation of resting cells is potentially undesirable for the production of ethanol at a large scale, the ability to form resting cells appears to hold some advantages for *C. thermocellum* survival, which have only just begun to be explored in this work.

## Materials and methods

### Organisms, substrates, and culture conditions

*Clostridium thermocellum* ATCC 27405 was used for all experiments. Before stress induction, *C. thermocellum* was grown overnight in 100 ml anaerobic serum bottles at 60°C in MTC medium [40] supplied with either 5 g/L cellobiose (Sigma) or crystalline cellulose (Avicel, PH105, FMC Corp., Philadelphia, PA) as the primary carbon source unless otherwise specified. All media contained 0.025% resazurin as a redox indicator and were purged with nitrogen before sterilization. A 10% transfer of overnight *C. thermocellum* culture was used to inoculate triplicate bottles of modified media with components added or omitted as described in the text in order to apply stress. Samples were examined microscopically every 8 hours, and it was determined that 24 h after induction was the most practical and consistent time point to quantify cells and resting forms. Stress conditions were all performed in bottles with Avicel as the carbon source unless otherwise noted. Growth medium modifications were made as follows: low phosphorous, potassium phosphate monobasic was eliminated from the media; low nitrogen, urea was eliminated from the media; no vitamins, vitamins were eliminated; added acetate, sodium acetate (Sigma) was added to the media before inoculation at the final concentration of 3 g/L; added ethanol, 200 proof ethanol (JT Baker) was added by%, v/v in quantities of 0.2%, 1%, 2%, 4% and 10% before inoculation; oxidative stress, sterilized air was added by%, v/v in quantities of 0%, 2%, 4%, 10%, 20%, 100%; substrate changes, cultures were first cultured on either 5 g/L cellobiose or 5 g/L Avicel. After 24 h of growth, a 10% transfer of each culture was made to media containing the other carbon source.

### Starvation conditions

In order to determine the effect of rapid starvation on the cells, cells were maintained in a continuous fermentor at a flow rate of 100 ml/h. The basic procedure was as follows: A 10 L carboy of MTC media was prepared. Solutions, vitamins and 3 g/L cellobiose were added by filtration through a 0.22uM filter (Millipore), and the carboy was purged with Nitrogen gas (Airgas). The carboy was used to fill a 1 L fermentor (Sartorius), which was then inoculated with 50 ml of an overnight *C. thermocellum* cellobiose grown culture. The culture was maintained at pH 6.8 ± 0.05 via addition of base (4 M KOH), temperature controlled at 55 °C, and stirred at 100 rpm while the headspace was continuously purged with nitrogen at a rate of approximately 1 ml/min.Growth was monitored by optical density (OD) at 600 nm and by the rate of base addition. Once the culture reached mid-exponential phase (OD_600_ = 0.4), the culture was continuously diluted at a rate of 0.1 h^-1^ with fresh media, while waste media was expelled from the fermentor to maintain a total volume of 1 L. The culture was maintained at a steady growth rate for 4 residence times, after which the continuous feed was stopped. Cells were sampled and observed under a microscope at different times thereafter to determine changes in morphology. Media samples were also analyzed via HPLC to determine cellobiose, acetic acid, lactic acid, and ethanol concentrations throughout. Viability of cells was determined 24 h after the feed was stopped via plating and determination of CFUs. To ensure culture purity, single colonies obtained from dilution plating were sequenced using 16 S rRNA universal primers 27 F (5’ – AGAGTTTGATCATGGCTCAG – 3’) and 1492R (5’ – GGTTACCTTGTTACGACTT – 3’).

### Spore/L-forms determination

To determine the number of spores or L-forms present in a culture after exposure to stresses, all cultures were observed microscopically. Spores, L-forms and cells were quantified by manual counts of 5 randomly selected fields. Numbers reported are indicative of the averages of these counts, and the specified error indicates the standard deviation of each biological replicate.

### Spore purification and storage

*C. thermocellum* 27405 was grown on MTC medium with 5 g/L Avicel for 24 h, and then a 10% transfer was made to MTC medium with 5 g/L cellobiose to generate a population of spores and cells. This culture was harvested after 24 h of growth. Spores were separated from vegetative cells by centrifugation and a modified HistoDenz (Sigma) gradient [41] prepared in a 15 ml conical tube (Fisher). Tubes were prepared with a 1 ml 100% v/v Histodenz gradient on the bottom followed sequentially by 1 ml gradients of 75, 50, and 25% Histodenz. After 1 ml of cell culture was added, each gradient column was centrifuged for 1 hour at 3000xg at room temperature in a Beckman Coulter Allegra 6R centrifuge. Microscopic examination revealed that phase bright spores and terminal endospores settled primarily in the 50% Histodenz fraction. This fraction was isolated and spores were then pelleted at 15,000 rpm for 30 minutes using a Beckman Coulter Avanti T-25 centrifuge. The spore pellet was then resuspended in 50 ml sterile water and allowed to settle overnight. The bottom few milliliters of this suspension were recovered and found to be highly enriched in spores with essentially no vegetative cells observed. Spores were then stored in sterile water at −80°C for later use.

### L-form purification and storage

L-forms were generated using the starvation procedure described above, and quantified microscopically by counting the number of L-forms and cells in 5 randomly selected frames and averaging these quantities. Values are reported as a percentage of L-forms over total cells, and the standard deviation is also reported. As more than 98% of all cells manifested the L-form morphology under these conditions, removal of the remaining 2% of vegetative cells (mostly appearing as broken cell debris) was not undertaken. L-form cells were harvested into anaerobic serum bottles and stored at −80°C with 20% glycerol until later use.

### Electron microscopy

TEM images were taken at 100 kV on a FEI Tecnai F20ST FEG, equipped with a digital camera (XR-41B; Advanced Micros-copy Techniques). Spores were observed in the presence of vegetative cells, while L-forms were prepared separately in order to minimize the number of procedures they were subjected to. Preparation of TEM samples was carried out at room temperature. All cell types were washed once in PBS and fixed in 2% Glutaraldehyde (GTA)/1% Paraformaldehyde (PF) in 0.1 M NaCacodylate buffer pH 7.4 (NaCAC).After fixing for 1 h, the 2% GTA/1% PF fix solution was removed and replaced with fresh fixative. Fixation continued for 24 h. Samples were then washed in NaCAC, postfixed in 1% osmium tetroxide (OsO4) for 2 h, and en-bloc stained in 1% uranyl acetate for 30 min. Samples were dehydrated in ethanol and embedded in LX112 resin. Thin sections were stained with 2% methanolic uranyl acetate for 15 min and Reynold's lead citrate for 3 min.

### Heat tolerance

To determine heat tolerance of the different resting cell types, cultures of each cell type were adjusted to 10^4^ cells/ml using a Petroff-Hausser cell counter 3900 (Hausser Scientific). Cells were plated for viable counts in modified DSM 122 broth [42] with the addition of 50 mM 3-(N-morpholino) propanesulfonic acid (MOPS) sodium salt and 3 g/L trisodium citrate (Na_3_-C_6_H_5_O_7_·2 H_2_O) in order to determine number of initial CFUs/ml before treatment. All experiments were conducted in an anaerobic chamber (Coy Laboratories, Grass Lake, MI). Each cell type was then divided into triplicate samples in 2.0 ml eppendorf tubes (American Scientific) and incubated at 100°C using a Digital Drybath incubator (Boekel) for 0, 0.5, 1, 5, 10, and 30 minutes, serially diluted after each time point and then plated to determine the number of surviving cells with a lower limit of detection of 10 CFU/ml.

### Growth recovery analysis

To determine the time frame needed for spores and L-forms to resume normal growth, growth for each cell type was measured at OD_600nm_. Each trial was performed in triplicate and used separately generated cell populations, L-forms, or spore stocks to ensure reproducibility. Cells in an OD range of 0.4-0.6 were considered mid-log phase, and cells that reached OD1.0 after peaking at OD1.4 were considered stationary phase. Pure cultures of each cell type were counted using a Petroff-Hausser cell counter, and adjusted to 10^6^ cells/ml in modified DSM 122 broth. All samples were then serially diluted and plated in modified DSM 122 broth with 0.8% agar to determine CFU/ml. To control for errors in cell counting caused by non-viable cells, all final calculations were based only on CFUs. All samples were diluted serially from 10^6^ CFU/ml to 10 CFU/ml in a sterile round bottom 96-well plate (Corning). Optical density was recorded at 600 nm using a PowerWave XS (BioTek) spectrometer operated in an anaerobic chamber. The plate was incubated at 55°C for the duration of the experiment, and was shaken every 30 seconds. OD_600_ was measured every three minutes. The duration of lag phase was evaluated based on the time needed to reach an OD_600_ of 0.1.

## Competing interests

LL is a stockholder in Mascoma Corporation, a biofuels company.

## Authors’ contributions

EM carried out all fermentations and growth study work, contributed to identifying sporulation conditions, and drafted the manuscript. JI contributed to identifying sporulation conditions and to drafting the manuscript. LL conceived of the study and participated in experimental design. All authors contributed to the design and interpretation of experiments, as well as to editing and revising the manuscript. All authors have read and approved the final manuscript.
